# An Intriguing Presentation of Epstein-Barr Virus-Associated Hemophagocytic Lymphohistiocytosis

**DOI:** 10.7759/cureus.9561

**Published:** 2020-08-05

**Authors:** Syed P Quadri, Nitesh K Jain, Brooke L Brandon, Harshit Modi, Hasnain Bawaadam

**Affiliations:** 1 Medicine, Aurora Medical Center, Kenosha, USA; 2 Critical Care Medicine, Mayo Clinic, Mankato, USA; 3 Medicine, Deaconess Midtown Hospital, Evansville, USA; 4 Critical Care, Aurora Medical Center, Kenosha, USA

**Keywords:** secondary hlh, ebv, covid 19, pancytopenia, high ferritin, cytokine storm syndrome, h score, secondary hemophagocytic lymphohistiocytosis, hemophagocytic lymphohistiocytosis (hlh)

## Abstract

Hemophagocytic lymphohistiocytosis (HLH) is an immune related clinical syndrome with protean manifestations, varying presentation, clinically complex, with diverse causes, and is an under-recognized entity which carries high morbidity and mortality. It is precipitated by an immunological trigger in a susceptible host resulting in immune activation and dysregulation leading to disruption of immune homeostasis, cytokine storm and multi-organ failure. We describe a case of Epstein-Barr virus (EBV) associated HLH with its typical diagnostic challenges and associated high mortality rate. Certain diagnostic criteria and online tools may help to arrive at an earlier presumptive diagnosis which, in turn, may expedite treatment and lead to better clinical outcomes.

## Introduction

Hemophagocytic lymphohistiocytosis (HLH) is an extremely rare and potentially life-threatening hematological disorder, characterized by clinical features of extreme inflammation and an unregulated immune system. It is precipitated by an immunological trigger in a susceptible host resulting in immune activation and dysregulation leading to disruption of immune homeostasis, cytokine storm and multi-organ failure. We describe a case of Epstein-Barr virus (EBV)-associated HLH with its typical diagnostic challenges and associated high mortality rate, but an early prompt diagnosis with appropriate treatment can lead to better outcomes. The objective of this clinical case report is to highlight the need for awareness and the complexity surrounding the diagnosis in an effort to lower subsequent morbidity and mortality.

## Case presentation

**Figure 1:**
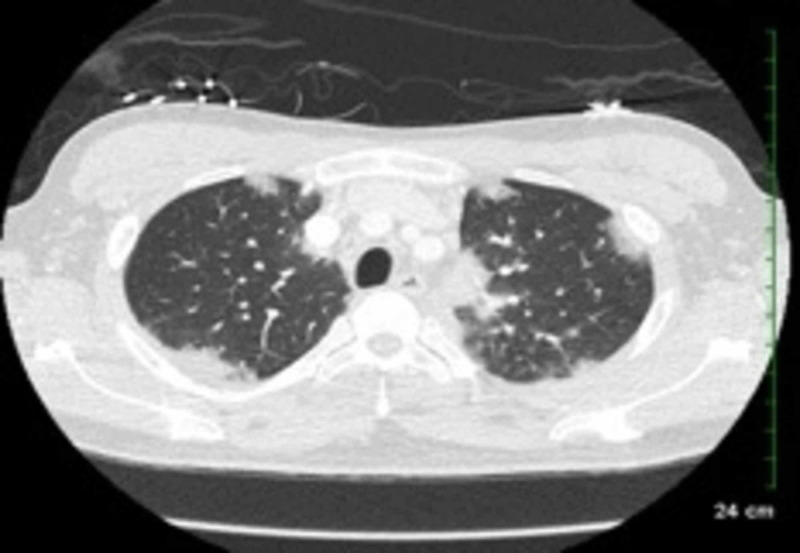
CT scan of the chest revealing bilateral lung infiltrates

A 43-year-old Caucasian male with no significant past medical history initially presented with severe fatigue, upper extremity joint pain, jaw pain, and new onset daily headaches. He was treated with a 10-day course of oral steroids for suspected temporomandibular joint dysfunction; this temporarily improved his symptoms. A few weeks later, his symptoms returned and he also developed daily high fevers with drenching night sweats. During this time, the patient was seen in the emergency room twice with dyspnea and cough and was treated for “presumed pneumonia” with broad spectrum oral antibiotics. He was eventually admitted to our hospital for failure of outpatient treatment of pneumonia. Computed tomography scan of the thorax showed bilateral ground glass opacities, with no evidence of mediastinal lymphadenopathy (Figure 1). He was noted to have pancytopenia, cervical lymphadenopathy and parotid gland swelling. EBV polymerase chain reaction (PCR) was elevated to 47,400 copies per ml. Ferritin was markedly elevated at 54,212 mcg/L; lactate dehydrogenase (LDH) was elevated to 2,125 U/L. He underwent parotid gland biopsy which was EBV-negative and demonstrated acute sialadenitis with necrosis. An extensive autoimmune, infectious and hematologic workup was equivocal. He was treated with broad spectrum IV antibiotics and high dose steroids. He remained afebrile for four days, and was ultimately discharged home on a two-week course of doxycycline, valacyclovir and fluconazole.

**Figure 2:**
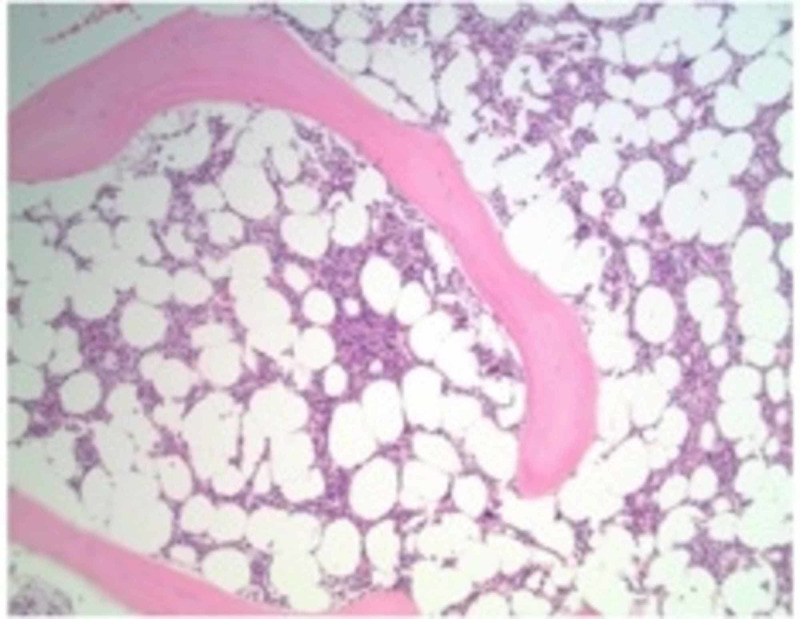
Bone marrow biopsy showing hypocellularity

His symptoms returned the following month with fevers, headaches and night sweats. The outpatient evaluation revealed a new non-tender, non-pruritic erythematous rash on his chest. Bone marrow biopsy was obtained which revealed a hypocellular marrow with trilineage hematopoiesis (Figure 2), negative for dysplasia; moderate number of Pelger-Huet type neutrophils; single lymphoid aggregate with an unremarkable flow cytometric profile. Subsequent positron emission tomography (PET)-scan demonstrated diffuse hypermetabolic lymphadenopathy in the mediastinum, upper abdomen and retroperitoneum, as well as pleural thickening and pulmonary hypermetabolic opacities. He was advised to obtain outpatient biopsy of the abdominal lymphadenopathy with interventional radiology, but he was unable to schedule it.

The patient was readmitted to the hospital few days later due to continued weight loss and seizure-like activity at home. CT head was unremarkable. He was found to have elevated liver enzymes with aspartate aminotransferase (AST) of 1,329 U/L, alanine aminotransferase (ALT) of 725 U/L and alkaline phosphatase (ALP) of 328 U/L. Liver biopsy revealed scattered acidophil bodies which can be seen in infections or drug-induced liver injury, rare EBV-positive cells but no evidence of fibrosis or lymphoproliferative disorder. He was once again found to have elevated ferritin of 35,732 mcg/L. High dose dexamethasone 40 mg/day was started for treatment of presumed HLH, based on labs and clinical presentation (Table 1). Chest X-ray showed right mid-lung opacity. The patient developed acute respiratory failure requiring intubation and mechanical ventilation. Bronchoscopy with lavage revealed Aspergillus species and he was started on appropriate anti-fungal treatment immediately. Repeat bone marrow biopsy was performed due to pancytopenia and demonstrated changes consistent with HLH, with definitive evidence of hemophagocytosis. Despite treatment, the patient continued to decline and developed rapid multi-organ failure. He was transitioned to comfort care and expired the same day.

**Table 1:** H-score A: H-score of 175 confers a 54-70% probability of hemophagocytic syndrome. B: H-score of 212 confers a 93-96% probability of hemophagocytic syndrome.

Parameter	Index hospitalization	Final hospitalization
Known underlying immunosuppression	No	No
Temperature	103.2 F	97.9 F
Hepatomegaly and/or splenomegaly	Yes	None
Number of cytopenias (1, 2 or 3 lineages)	3	3
Ferritin (ng/ml)	54,212	35,732
Triglycerides (mg/dL)	114	180
Fibrinogen (mg/dL)	Not recorded	126
Aspartate aminotransferase (AST) (U/L)	1329	888
Hemophagocytosis features on bone marrow aspirate	No	Yes (at autopsy)
H-score	175^A^	212^B^

Autopsy results and analysis

The major finding in the patient’s autopsy was hemophagocytic histiocytosis with rare EBV-positivity noted in the bone marrow analysis, suggesting that the HLH was due to EBV infection. There was no evidence of any lymphoproliferative disorder. Lung examination revealed acute Aspergillus spp. pneumonia with left upper lobe abscess formation, likely related to his immunocompromised state in the setting of HLH. Also discovered was multi-organ vasculitis, the significance of which is unclear. Although there have been rare reported cases of HLH in the setting of vasculitis, the laboratory evidence of EBV infection and finding of rare EBV-positive cells in the bone marrow on autopsy and in the ante-mortem liver suggest that the HLH was related to EBV infection. The official cause of death in the patient was determined to be pulmonary aspergillosis with organizing pneumonia in the setting of hemophagocytic lymphohistiocytosis, likely secondary to Epstein-Barr virus infection.

## Discussion

HLH is a rare life-threatening condition characterized by hyperinflammation and immune system dysregulation [1]. It can occur in both familial and acquired forms [2]. Acquired HLH is associated with various infections including bacterial, viral, fungal and parasitic; autoimmune diseases including vasculitis, rheumatological disorders, malignancy, and acquired immune deficiency states including AIDS, and with drug use [3,4].

The basic pathophysiology in HLH is that the natural killer (NK) cells and cytotoxic T-lymphocytes, which represent the innate and acquired immunity respectively, have altered cytotoxic function. This is coupled with excessive macrophage activation. The NK cells and lymphocytes are not able to eliminate the macrophages by perforin-mediated cytotoxicity. Eventually there is a huge amount of cytokine release by all these above-mentioned immune cells resulting in excessive tissue damage across multiple organ systems [1,2]. Toll like receptor activation of the immune system can also be another potential mechanism of HLH [5].

It usually presents with fevers, hepatosplenomegaly, pancytopenia along with elevated liver enzymes, significant ferritin elevation and hypertriglyceridemia [6]. EBV is the most common infection associated with acquired HLH [7]. Other viruses such as adenovirus and cytomegalovirus can also trigger HLH. Infectious triggers cause immune activation. Alternatively, immune deficiency can also trigger HLH such as HIV disease, malignancy, and certain rheumatological diseases [1].

Diagnosis of HLH is challenging and requires a high index of suspicion. It is not uncommon for patients to have had a prolonged or multiple hospitalizations associated with clinical decline in health status before the diagnosis is finally made. Diagnostic criteria have been formulated as per the HLH-2004 protocol which includes molecular, clinical and laboratory criteria [6]. Genetic testing should be performed in all suspected cases. The HLH-2004 protocol has not been validated in adults in secondary HLH. Also, it has practical difficulty in application as the various tests required for completion of the diagnostic protocol take time and are not rapidly available.

In one study of 369 patients, the clinical findings included in HLH-2004 protocol were found to variably distributed in the patient population [8]. The prevalence of low or absent NK cell function was 71%. Prolonged fever was seen in 95% of the population, whereas depleted cell lines were seen in up to 92% cases. Splenomegaly was seen in 89% of the cases. Hypertriglyceridemia or hypofibrinogenemia were noted in 90% cases. Elevated ferritin greater than 500 mcg/L was found in 94% of the patients. In contrast, evidence of hemophagocytosis on bone marrow exam was only seen in about 82% of the study subjects, the least prevalent of the markers. Markers of increased T cell activation such as elevated CD25 counts were seen in up to 97% of the patients.

It is therefore to be noted that the characteristic finding of “Hemophagocytosis” in the liver, lymph nodes or bone marrow is not necessary for diagnosis, however, its presence can aid in diagnosis. Flow cytometry in a specialized laboratory can be very helpful with immunological markers [8].

The H-score is a much more pragmatic and easier tool to apply by the patients’ bed side and can help with rapid diagnosis and therefore help with treatment considerations, as timely intervention can help improve prognosis. An online calculator is also available for calculating the H-score [9,10].

HLH should be considered in the differential diagnosis of any patient presenting with unexplained fever, pancytopenia, hepatosplenomegaly, rash, lymphadenopathy, neurologic symptoms, liver function abnormalities and high serum ferritin. Elevated ferritin due to macrophage activation is helpful in children but much less specific in adults. However, a very high elevation of ferritin and sCD25 are very helpful in making the diagnosis.

Treatment should be initiated in a timely manner and consists of supportive care, treating the underlying triggers like infection, immunosuppressive agents, monoclonal antibodies, cytotoxic therapy and biological agents [11-14]. Hematopoietic stem cell transplant may be an option for definitive treatment of HLH in selected patients [15]. Delay in diagnosis is associated with poor prognosis due to multi-organ involvement. Mortality rate remains high despite treatment, ranging from 18-24% in EBV-associated HLH [16].

Given the current day pandemic, severe cases of COVID-19 can act as HLH mimic, as the former manifests as severe sepsis with cytokine storm syndrome. This needs further elucidation and some therapies like IL-6 inhibitor tocilizumab, which have been used for HLH/cytokine storm syndrome previously, are being tried as therapy for COVID-19 [17]. Several academic institutions have employed the H-score mentioned above to guide therapeutic decisions on IL-inhibitors. An H-score greater than 250 confers a 99% probability of HLH and a score of less than 90 confers a less than 1% probability of HLH [18, 19].

## Conclusions

Our case highlights the importance of having a high index of suspicion for HLH in unusual presentations of febrile illness with pancytopenia, even in otherwise healthy individuals. It is possible that our patient could have benefited from earlier treatment with steroids or other appropriate agents, rather than later in the course of the disease. Further research is needed to better understand the pathophysiology of HLH at a molecular level, which could lead to modification of existing treatment protocols and improve patient survival.
